# Impairments in Episodic-Autobiographical Memory and Emotional and Social Information Processing in CADASIL during Mid-Adulthood

**DOI:** 10.3389/fnbeh.2014.00227

**Published:** 2014-06-25

**Authors:** Angelica Staniloiu, Friedrich G. Woermann, Hans J. Markowitsch

**Affiliations:** ^1^Physiological Psychology, University of Bielefeld, Bielefeld, Germany; ^2^Hanse Institute of Advanced Science, Delmenhorst, Germany; ^3^MRI Unit, Bethel Epilepsy Center, Bielefeld, Germany; ^4^Center of Excellence “Cognitive Interaction Technology” (CITEC), University of Bielefeld, Bielefeld, Germany

**Keywords:** chromosome 19, gene mutation, episodic memory, problem solving, cognitive flexibility, social information processing

## Abstract

Cerebral autosomal dominant arteriopathy with subcortical infarcts and leukoencephalopathy (CADASIL) – is the most common genetic source of vascular dementia in adults, being caused by a mutation in NOTCH3 gene. Spontaneous *de novo* mutations may occur, but their frequency is largely unknown. Ischemic strokes and cognitive impairments are the most frequent manifestations, but seizures affect up to 10% of the patients. Herein, we describe a 47-year-old male scholar with a genetically confirmed diagnosis of CADASIL (Arg133Cys mutation in the NOTCH3 gene) and a seemingly negative family history of CADASIL illness, who was investigated with a comprehensive neuropsychological testing battery and neuroimaging methods. The patient demonstrated on one hand severe and accelerated deteriorations in multiple cognitive domains such as concentration, long-term memory (including the episodic-autobiographical memory domain), problem solving, cognitive flexibility and planning, affect recognition, discrimination and matching, and social cognition (theory of mind). Some of these impairments were even captured by abbreviated instruments for investigating suspicion of dementia. On the other hand the patient still possessed high crystallized (verbal) intelligence and a capacity to put forth a façade of well-preserved intellectual functioning. Although no definite conclusions can be drawn from a single case study, our findings point to the presence of additional cognitive changes in CADASIL in middle adulthood, in particular to impairments in the episodic-autobiographical memory domain and social information processing (e.g., social cognition). Whether these identified impairments are related to the patient’s specific phenotype or to an ascertainment bias (e.g., a paucity of studies investigating these cognitive functions) requires elucidation by larger scale research.

## Introduction

In 1991, a French group (Tournier-Lasserve et al., 1991) described the occurrence of an “[a]utosomal dominant syndrome with strokelike episodes and leukoencephalopathy” (Title of the publication). They studied 45 family members and found in 9 of them transient ischemic attacks and strokes, together with widespread involvement of the white matter. Two years later they described autopsy findings of one of the nine patients – a woman who died at age 59 (Baudrimont et al., 1993). When this working group in 1993 coined the acronym cerebral autosomal dominant arteriopathy with subcortical infarcts and leukoencephalopathy (CADASIL) for this disease condition, they stated that related cases had been published since 1977 (Tournier-Lasserve et al., 1993). In a more recent review, Chabriat et al. (2009) remarked that the first case of CADASIL might have been described in 1955 in two sisters by van Bogaert (1955) and categorized at the time as Binswanger’s disease.

Cerebral autosomal dominant arteriopathy with subcortical infarcts and leukoencephalopathy is regarded as a hereditary, autosomal dominant “disease with high penetrance in which occlusion of small arteries in the brain of adults results in small deep brain infarcts and progressive accumulation of demyelination areas in the brain” (André, 2010, p. 287). Intrafamilial phenotypic variability has been reported. Phenotypic variations in monozygotic twins were identified and attributed to environmental factors and epigenetic effects (Dichgans et al., 1998; Mykkanen et al., 2004, 2009). *De novo* mutations and homozygous cases were also infrequently described (Chabriat et al., 2009). A mutation in the NOTCH3 gene in chromosome 19 apparently first leads to a microangiopathy of the small arteries supplying the brain, and – as a consequence – ultimately to dementia (Joutel et al., 1996). Magnetic resonance imaging (MRI) sometimes shows neural abnormalities from about age 30 onward, strokes may manifest themselves from about age 35 onward, mood disorders after age 40, and dementia may develop between ages 50 and 60 (Sabbadini et al., 1995; Chabriat et al., 2009; André, 2010). Seizures may happen in 5–10% of the patients (Chabriat et al., 2009). Neuroimaging data typically include multifocal white matter lesions, which affect (anterior) temporal lobe and external capsula (Chabriat et al., 1995; Joutel et al., 1996; Brass et al., 2009; Jacqmin et al., 2010; Epelbaum et al., 2011). Changes in basal ganglia, thalamus, brainstem and corpus callosum, and frontal white matter can also be detected (Brass et al., 2009; Chabriat et al., 2009). There is no effective disease modifying treatment available (Dichgans et al., 2008).

Cognitive impairments are considered to be the second most common manifestation of CADASIL. Several studies have linked gene defects to changes in cognition; however, the relation between genotype and brain development and genotype and cognition is very complex. This might also hold true for aberrations related to chromosome 19 (Grimwood et al., 2004; Van der Aa et al., 2010). In patients with CADASIL, the development and progression of changes in cognition have prevailingly been connected to the occurrence of recurrent strokes, but other mechanisms may play a role (Amberla et al., 2004; Peters et al., 2005).

Executive dysfunctions, which were reported to already be present in patients with CADASIL of age 35–50 years, and alterations in processing speed belong to the initial cognitive changes (Peters et al., 2005; Chabriat et al., 2009). Other cognitive impairments, which have been described, are deficits in attention and concentration, visuo-spatial skills, language, verbal and visual memory, and reasoning (Chabriat et al., 2009). Performance on recognition tasks is usually relatively or partially preserved. According to our knowledge, there are no accounts of formal assessment of the retrograde episodic-autobiographical memory in patients with CADASIL with the autobiographical memory interview (Kopelman et al., 1990) or its variants. Most studies which were carried out in patients with CADASIL used testing paradigms, which corresponded to old views of episodic memory (e.g., testing of memory for lists of pairs of words) (Tulving, 1972). Some authors stated that autobiographical memory is “typically impaired from the early stages of Alzheimer’s disease (AD) and vascular dementia” (Naylor and Clare, 2008, p. 591). Others argued that “in comparison with AD patients, those with a diagnosis of vascular dementia display a relative preservation of episodic memory” (Peters et al., 2005, p. 2078). One study reported impairments in facial affect recognition in patients with CADASIL (Valenti et al., 2013). Their sample of patients had a higher rate of co-morbid depressive symptoms, which may have confounded the findings. Furthermore, no other complex tests of face-emotion processing were carried out and no tests on social cognition were performed. Overall there is a dearth of data in the CADASIL population on the ability for social information processing (Adolphs, 2010), namely social *perception* (e.g., face perception), *social cognition* (theory of mind, empathy, social judgment), and *social regulation* (emotion regulation, monitoring/error correction, self-reflection, deception).

Herein, we aim to impart novel insights into the nature and extent of changes in the episodic-autobiographical memory and social information processing in CADASIL, by reviewing the neuropsychological profile of a patient with a diagnosis of CADASIL, possibly due to a *de novo* mutation in NOTCH3 gene, and a strong premorbid educational and occupational achievement, who became symptomatic in his early to mid forties.

## Case Report

SP is a 47-year old man who self referred to our facility for a neuropsychological assessment, several months after he had received a diagnosis of CADASIL. Apart from a long standing problem with color perception and a questionable history of meningoencephalitis in childhood, SP had reportedly been in good health until his early 40s, when he out of the blue experienced an episode of diplopia (double-seeing). SP sought medical advice. He received a diagnosis of cranial mononeuropathy with left trochlear nerve (IV) paresis of unclear etiology, and a brief treatment with glucocorticoids. The diplopia remitted after a couple of weeks. Serum and cerebrospinal fluid (CSF) laboratory results were negative. *Borrelia* serology was also interpreted as unremarkable at the time. X-rays of the thorax showed no abnormalities. A head computed tomography (CT) demonstrated no evidence of intracerebral bleeding, but pointed to modifications in prefrontal white matter. MRI provided no evidence of infarcts or tumors. It evidenced changes in the white matter, namely in the periventricular areas and corpus callosum. The brain changes were judged as being old at the time and attributed to a questionable history of meningoencephalitis in childhood. Two years later, SP experienced a recurrence of the diplopia, which was linked to unilateral abducens nerve (VI) palsy. While the etiology of the sixth nerve palsy could not be ascertained, the seemingly progressive white matter abnormalities detected by structural MRI raised at the time the suspicion of multiple sclerosis and prompted a comprehensive work up. The diplopia recovered after approximately 2 weeks after another brief trial of glucocorticoid anti-inflammatories. SP’s symptoms however broadened. He developed word finding difficulties, dizziness, and problems with orientation. Later both complex focal seizures and grand mal seizures occurred. Thorough medical and laboratory investigations were carried out and several therapeutic trials were initiated. SP stopped driving an automobile and stated that he has not driven any since his diagnosis of seizures. Until CADASIL was proven by genetic studies, SP’s working diagnoses encompassed multiple sclerosis, neuroborreliosis (Lyme’s disease), viral encephalitis, a mitochondrial disease with leukoencephalopathy, a hereditary metabolic leukoencephalopathy, and a cerebral amyloid angiopathy. Serological screening testing with enzyme-linked immunosorbent assay (ELISA) was positive for IgG antibodies against *Borrelia burgdorferi* (markers of previous infection), but negative for IgM antibodies (markers of actual infection). Confirmatory Western immunoblot tests followed. They were negative for IgM antibodies against *B. burgdorferi*, but IgG blots yielded positive results. The CSF evaluations provided evidence of positive IgG antibodies against *B. burgdorferi*, but were again negative for IgM antibodies. CSF to serum anti-*B. burgdorferi* antibody index was however mildly elevated. CSF analysis was positive for oligoclonal IgG bands Type III, but revealed no proteinemia or pleocytosis or abnormalities in glucose and lactate levels. SP had no meningitis signs or symptoms. Serum cell blood counts and differentials, liver and renal function tests were within normal limits. Despite insufficient and debated evidence for active neuroborreliosis, SP received a 21-day parenteral course of a cephalosporin antibiotic (Ceftriaxone 2 g a day), with unclear benefits. For the control of seizure activity, treatment with sodium divalproate was begun, with good seizure control and no significant subjective untoward effects. Contradictory findings of oligoclonal bands on the protein electrophoresis of CSF, a negative family history and progressive brain abnormalities affecting the white matter maintained for a substantial period the suspicion of multiple sclerosis (Zeman et al., 1993; Dichgans et al., 1999; Compston and Coles, 2008; Bentley et al., 2011). In the face of repeated and additional laboratory investigations the diagnosis of multiple sclerosis was subsequently discarded. Several factors strongly argued against viewing the infection with *B. burgdorferi* as a primary mechanism for the cognitive and physical changes of SP (Yamamoto et al., 2011; Eikeland et al., 2012), in addition to the genetic testing (see below). As mentioned above, SP received an adequate, evidence-based treatment with an antibiotic that is very active against Lyme borreliosis and crosses the blood–brain barrier (Stanek et al., 2012), despite questionable support for an active diagnosis of neuroborreliosis. There are indeed authors who argue that positive *B. burgdorferi* immunological markers in CSF may not mean that the individual has active neuroborreliosis (Roos and Berger, 2007). They reportedly could represent proof of a previous infection (Stanek et al., 2012) or leakage of antibodies from serum across the blood–brain barrier, in the context of a CADASIL-related increase in the permeability of the blood–brain barrier (Roos and Berger, 2007; Collongues et al., 2012). Furthermore, asymptomatic infection with *Borrelia* may occur relatively frequently in Europe (Steere et al., 2003). Additionally, the existence and magnitude of chronic courses of neuropsychological deficits after symptomatic *B. burgdorferi* infection are debated (Shadick et al., 1999; Eikeland et al., 2012; Stanek et al., 2012). Some authors reported that neuropsychological deficits pertaining to memory and executive functions may follow a chronic course (Eikeland et al., 2012) in a subset of patients, lasting for more than 6 months (post-Lyme borreliosis syndrome) (Stanek et al., 2012). Shadick et al. (1999) found that subjective memory impairments were reported by patients with a history of Lyme disease 6 years after the infection. However, performance on objective memory tests was deemed in this study as being comparable with that of matched healthy participants without a history of Lyme disease. Finally, although brain changes may occasionally persist after successful antibiotic treatment in neuroborreliosis (Agarwal and Sze, 2009; Hildenbrand et al., 2009), SP’s pattern of brain changes had several unique elements that supported the final diagnosis of CADASIL (Chabriat et al., 2009) (see below). It was therefore concluded that the characteristics of the patient’s features mandated consideration of other diagnostic entities.

Blood work results were remarkable for mildly increased homocysteine levels, slightly increased sodium levels, slightly increased hematocrit, low folic acid level, borderline low vitamin B_12_ levels, slightly increased glucosylated hemoglobin, and evidence of mild dyslipidemia. Cholesterol fractions HDL and LDL reached normal limits with lipid lowering medication (a statin). Serology for vasculitis and autoimmune diseases (systemic lupus erythematosus) and lues and human immuno deficiency virus (HIV) was negative. There was no indication of infection with cytomegalic virus, herpes simplex virus, *Toxoplasma gondii*, or active infection with varicella zoster. Thyroid function tests and ceruloplasmin plasma levels were within normal limits. Investigation of AD serum and CSF biomarkers yielded unremarkable results (Jahn, 2013). Due to suboptimal vitamin B values, replacement with both vitamin B_12_ and folic acid was instituted. An angiotensin converting enzyme inhibitor (ACEI) was apparently started for borderline blood pressure values, with good results. Electrocardiogram (ECG) studies revealed a normal sinus rhythm. Several electroencephalograms (EEG) were carried out. The most recent EEG with hyperventilation test was unremarkable. Electrophysiological investigations of tibial, peroneal, sural, and median nerves did not yield any significant results. Electromyography was unremarkable, with the exception of some unspecific changes of the right vastus lateralis muscle. The neuro-ophthalmology exam was suggestive of a sub-clinical optic nerve (II) neuropathy with bilateral pathological visual evoked potentials as well as bilateral temporal pallor of the papilla. Incidentally, optic nerve changes of ischemic origin in CADASIL were described in some case reports (Rufa et al., 2005) and delays in the visual cortical responses were also presented in case series (Parisi et al., 2000, 2003). Auditory evoked potentials were on the left side mildly slowed down, consistent with a change in the pontine area. Interestingly, abnormal brainstem auditory evoked potentials were also depicted in a member of one Italian family with CADASIL by Burganza et al. (1996). In the case of SP, the somato-sensory evoked potential of the median nerve was unremarkable.

Genetic testing was performed and confirmed an Arg133Cys mutation in the NOTCH3 gene, which has been linked to CADASIL in previous reports (Mykkanen et al., 2004, 2009). Given that both biological parents of SP were healthy and well above 71 years (the oldest age when CADASIL was ever reported to first manifest itself clinically) and that there was no clear evidence of a family history of CADASIL, a spontaneous *de novo* mutation was suspected (Joutel et al., 2000; Coto et al., 2006; Bentley et al., 2011; MacArthur et al., 2014). The results of genetic testing, neuroimaging, and the constellation of signs and symptoms supported the final diagnosis of CADASIL.

## Materials and Methods

### Neuroimaging data examination

Several imaging investigations were performed, such as CT scan of the brain (as described above), repeated head MRIs, ultrasound, and angiography. Extracranial and transcranial duplex ultrasound evidenced a normal vertebrobasilar artery and lack of significant changes in common carotid artery and external and internal carotid arteries. Magnetic resonance angiography of the head and neck yielded no significant abnormalities.

**Figure 1:**
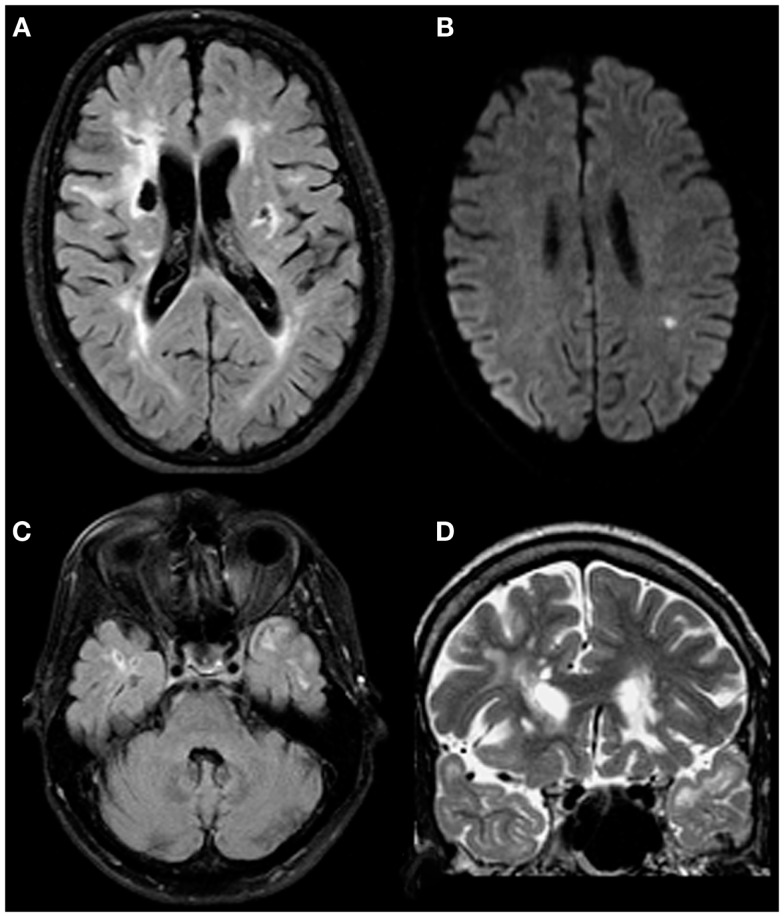
**Both chronic and acute changes in a patient with proven CADASIL**. Chronic lacunar defects and gliotic changes affecting bilateral fronto-parietal regions including basal ganglia on both sides [**(A)**; axial FLAIR image]. Subacute lacunar infarct in the left parietal white matter [**(B)**; axial diffusion weighted image]. Gliotic changes affecting both anterior temporal lobes are thought to be typical for CADASIL [**(C,D)**; axial FLAIR and coronal T2 weighted images; please note: lacunar defects in the right temporal lobe are adjacent to the right-sided amygdala].

T1 and T2 sequences of his central nervous system were obtained with a conventional 1.5-T MRI device. Earlier scans pointed to abnormalities in periventricular white matter, corpus callosum, and centrum semiovale (Chabriat et al., 2009). Later, multiple lacunar lesions were described bilaterally in the basal ganglia and in the left frontal region. Abnormalities were also reported in the brainstem and signs of microbleeds were detected in the thalamus. The most recent neuroimaging exam (which was performed 1 month prior to the present neuropsychological examination) underlined the presence of chronic lacunar defects and gliotic changes affecting bilaterally fronto-parietal regions and basal ganglia. Subacute lacunar infarcts were visualized in the left parietal white matter in addition. Lacunar defects at this time were also identified in the right temporal lobe and adjacent to the right amygdala. Gliotic changes affecting both anterior temporal lobes were also visualized. Figure 1 illustrates the most relevant changes.

### Anamnesis: Personal and family history

SP denied a history of alcohol or drug use. He reported several environmental allergies. The last neurological exam, performed several months before the assessment was significant for a left light facial paresis.

At the time of the neuropsychological assessment, SP was taking aspirin, a hypolipidemic drug (a statin), an ACEI drug for hypertension, a proton pump inhibitor medication for gastro-esophageal reflux, sodium divalproate for seizure protection, folic acid, and vitamin B_12_. He reported tolerating well the medication and that both his seizures and blood pressure were well controlled on the current regimen. Last valproic acid trough blood levels were within therapeutic range.

SP’s family history was significant for a sibling suffering from a hearing deficit with adult onset, of unclear origin (questionable acoustic neurinoma). Although cranial neuropathies including involvement of nerve VIII have occasionally been reported in patients with CADASIL, according to SP’s knowledge his sibling’s condition was stable and unremarkable for other symptoms. Parents were in their 70s and healthy according to SP’s knowledge. SP described his birth and postpartum development as uneventful. According to his knowledge, he achieved developmental milestones at a normal age. SP reported being involved in a long-term romantic relationship. He was holding for several years an important and rewarding academic position in business and economy, but was faced with the prospects of a premature forced retirement since he had received the diagnosis of CADASIL. SP appeared still confident in his intellectual abilities and judged himself as being fit for his profession. Although SP had undergone other neuropsychological assessments in the past, he wanted to have a second opinion and therefore contacted our department for a new evaluation.

### Informant history

Informant history was obtained from SP’s partner and from reviewing copies of consultants and health care provider reports, which SP made available to us. SP’s partner did not elaborate on SP’s difficulties, but opined that SP was still able to deal with the demands of his work, as they substantially relied on routines. SP alluded to some interpersonal difficulties with the partner that led to a temporary separation after his diagnosis with CADASIL, but did not give further details. In previous reports, it was remarked that SP had encountered difficulties at work since his diagnosis of CADASIL, where people had viewed him as scattered and questioned his professional competence. SP disagreed with these voiced concerns and stated that people questioned his work ability for strategic reasons. He perceived himself as being more patient than prior to his diagnosis. He denied problems with memory, although he admitted to engaging in computerized cognitive (including memory) training regularly. Health providers documented a reduction in affect range, mimic and gesturing, and voice monotony, but no conspicuous problems with personal boundaries. They noted that SP described having close social contacts with some co-workers, but no other regular social contacts.

### Neuropsychological examination

For the purpose of the assessment, SP was interviewed and evaluated with several tests. SP gave informed written consent for the participation in the assessment and study and publication of the report. The study adhered to the declaration of Helsinki.

SP had a 1-day appointment in our unit, during which a substantial number of neuropsychological tests were administered. SP did not recall or report the nature of neuropsychological tests he had undergone in the past; however, the copies of health care providers reports indicated that he had at least one evaluation with consortium to establish a registry for AD (CERAD) test battery, more than 3 months prior to our meeting (Morris et al., 1988). This battery contains: mini-mental state examination (MMSE) (Folstein et al., 1977), the Trail Making Test part A and B, a test for phonetic fluency, an abbreviated version of the Boston naming test (15 items), a verbal fluency (animal category) test, a verbal memory test (word list), and a visual memory test and visuo-spatial skill tests.

The tests that were applied in our department were the following:

- Tests for handedness and brain lateralization: The lateral preference inventory (LPI) for measurement of handedness, footedness, earedness, and eyedness (Ehrenstein and Arnold-Schulz-Gahmen, 1997).
- Tests for the estimation of intelligence and overall cognitive status: Abbreviated Wechsler Adult Intelligence Test-Revised (Block test and Picture Completion test) (Dahl, 1972). MWWT-B or Mehrfachwahl-Wortschatz-Intelligenztest-B (Lehrl, 2005), a German version of the National Adult Reading Test NART (Nelson, 1982); DemTect, a dementia screening instrument consisting of six verbal and numerical subtests (Calabrese and Kessler, 2000); Mini-Mental-State-Examination (Folstein et al., 1977; Kessler et al., 1990).
- Tests for the evaluation of attention, concentration, and processing speed: Trail Making Test A and B (TMT-A + TMT-B; Lezak, 1995; Reitan, 1958); d2-Test (Brickenkamp and Zillmer, 1998); Attention Index of the German version of the Wechsler memory scale-revised (WMS-R; Härting et al., 2000).
- Tests for short-term memory and working memory: Wechsler memory scale-revised; digit span and block span forward and backward.
- Test for the evaluation of constructional functions and planning: Copy administration of the Rey–Osterrieth Figure Test (Osterrieth, 1944; Lezak, 1995).
- Tests for the evaluation of the verbal and non-verbal explicit anterograde long-term memory: Wechsler memory scale-revised (Härting et al., 2000); verbal learning memory test (VLMT) (Helmstaedter et al., 2001); Rey–Osterrieth-Figure (copy trial followed by delayed recall after 30 min) (Lezak, 1995); Doors Test of the Doors and People Test (Baddeley et al., 1994); Emotional Pictures Test (Cramon et al., 1993).
- Tests for the evaluation of retrograde memory: Extended autobiographical memory interview (Seidl et al., 2006), a German language adaptation of the autobiographical memory interview of Kopelman et al. (1990); Famous Faces Test (Fujiwara et al., 2008).
- Test for the evaluation of procedural memory: Mirror reading test (Cramon et al., 1993; Borsutzky et al., 2008, 2010). For the mirror reading test the variant that is presented by Borsutzky et al. (2008, 2010) was applied.
- Tests for the assessment of executive functions, including problem solving, cognitive flexibility: TMT-B (Lezak, 1995); Tower of Hanoi (Borys et al., 1982; Spitz et al., 1982; Lezak, 1995); California Card Sorting Test (Delis et al., 1992); problem solving test of Cronin-Golomb et al. (1987a,b); Wisconsin Card Sorting Test (Nelson, 1976).
- Tests of verbal fluency: Controlled Oral Word Association Test (COW; Golden et al., 2002); Supermarket task (word production; subtest of the DemTect); Boston naming test (Kaplan et al., 1983; Lezak, 1995; Spreen and Strauss, 1998; Golden et al., 2002).
- Tests for evaluation of malingering tendencies: Test of memory malingering (TOMM; Tombaugh, 1996; Teichner and Wagner, 2004; Greiffenstein et al., 2008); Rey 15-Item-Test (Lezak, 1995).
- Tests for evaluation of emotional processing: Florida Affect Battery (translated as Tübingen Affect Battery; Bowers et al., 1991; Breitenstein et al., 1996); Emotional Pictures Test (Cramon et al., 1993).
- Tests for mood, personality, and psychopathological and psychological load screening: Beck depression interview (BDI-II; Hautzinger et al., 2006); the symptom checklist revised or SCL-90R (Hessel et al., 2001); Freiburg-personality-inventory-revised (FPI-R; Fahrenberg et al., 2001). The SCL-90R assesses psychiatric symptom load and psychological distress. It contains nine subscales, such as somatization, obsessive–compulsiveness, interpersonal sensitivity, depression, anxiety, anger–hostility, phobic anxiety, paranoid thinking, and psychoticism. The general psychological distress level is appraised based on a global severity index, which is derived from all subscales. The FPI-R offers an assessment of personality across 12 dimensions: life satisfaction, social orientation, motivation to achieve, inhibition, excitability, aggressiveness, stress, physical complaints, health worries, openness, extraversion, and neuroticism. Furthermore, we gave SP a self-rating questionnaire in which he had to estimate how much his personality has changed from the time prior to the CADASIL diagnosis to the present (Crook and Larrabee, 1990; Fast and Fujiwara, 1999).
- Tests for social information processing (face-emotion perception, social cognition, risk-taking behavior): German adaptation of the Reading the Mind in the Eyes Test (Baron-Cohen et al., 2001; Reinhold and Markowitsch, 2007); Multiple-Choice-ToM-test (MCTT; Adenauer et al., 2005); Florida Affect Battery; Bowers et al., 1991); Game of Dice Test (Brand et al., 2005; Brand and Markowitsch, 2010). The Multiple-Choice-ToM-Test (Adenauer et al., 2005; Staniloiu et al., 2013) version that we used demanded the patient to read 16 short stories. (The test is available in two variants, one with 30 short written stories and another one with 16 stories.) After each story the patient had to infer the mental states of a protagonist of the story by making use of a forced multiple choice layout with four answering alternatives (but only one right answer). The multiple choice configuration allows distinguishing three different types of faults: (a) mental states inferences that are “excessive”; (b) mental states inferences that are “too positive”; (c) selection of the distractor answer that indicates a neutral answer (i.e., a non-mental state inference such as physical causation).

The game of dice task (Brand et al., 2005; Brand and Markowitsch, 2010) is a fictitious gambling setting, presented via computer, with explicit rules for virtual gains, losses, and winning stratagems. It evaluates decision-making under risk.

## Results

### General behavioral observations

SP was cooperative, casually, but appropriately dressed, with good eye contact and fully oriented. He demonstrated willingness to take part in all tests and showed consistent effort to perform. He reported having some problems with concentration when too much interference was present. He subjectively appraised his memory as being good. He denied problems with mood, apart from some anxiety feelings related to the future of his job. He voiced hope that the results of the tests would indicate that he still was fit to work in his profession. He answered readily all questions and worked without measurable or notable tendencies to worsen, distort or falsify his performance. He promptly followed instructions. SP was clinically unimpaired with respect to audition, but had, according to his report, a long standing history of a color perception deficit. The latter however did not appear to interfere in a conspicuous way with his ability to recognize colors on the applied tests or subtests that used colored items, such a subtest of WMS-R (Härting et al., 2000), the Doors Test of the Doors and People Test (Baddeley et al., 1994), or the Wisconsin Card Sorting Test.

Below we present and discuss the results of the tests that SP undertook over a period of about 7 h (with breaks). A summary of the findings of SP’s evaluation is provided in Table 1.

**Table 1:** **Summary of neuropsychological testing results**.

Test/questionnaire	Score	Interpretation
**LATERALITY**		
Laterality preference inventory	15 right, 1 left	Right lateralized
**QUESTIONNAIRES**		
Self-evaluation prior to disease vs. present	SP only responds to parts of the questionnaire. He writes to be severely afraid of losing his job position, that his eating habits and hobbies have somewhat changed. He also noted changes in the relation with his partner, his ability to concentrate and act independently and that his interest in his disease has strongly increased	
Beck depression inventory	0	No tendency toward depression
Freiburg personality inventory	Stanines between 2 and 6	No serious conspicuousness
SCL-90-R	GSI: 0.38; PST: 24; PSDI: 1.375	Normal
**ATTENTION AND CONCENTRATION**		
Trail making test A + B	A: 43 s, 0 errors; B: 68 s, 0 errors	Average
d2-R test	75, 58 Omissions; PR < 1%	Massively deficient
WMS-R, attention and concentration	71	Massively deficient
**INTELLIGENCE AND GENERAL INTELLECTUAL STATUS**		
Mehrfach-Wahl-Wortschatz-test B (a test to estimate verbal IQ)	34 out of 37	High verbal IQ (130)
Abbreviated Wechsler intelligence test	Verbal: 22, 23; non-verbal: 12, 18	Verbal IQ above average, non-verbal subaverage
DemTect	10.5	“Mild cognitive impairment”
MMSE	27 Points	Borderline score which is, however, very low, given his intellectual background and age
**VISUOCONSTRUCTIVE ABILITIES**		
Rey–Osterrieth figure (ROF), copy	36 Points	Normal
**SHORT-TEM AND WORKING MEMORY**		
Digit span forward	5	Normal
Digit span backward	4	Subaverage
Block span forward	4	Impaired
Block span backward	4	Subaverage
**ANTEROGRADE MEMORY**		
ROF, reproduction after 1/2 h delay	4 Points	Severely impaired
WMS-R, general memory	78	Significantly below average
WMS-R, verbal memory	83	Significantly below average
WMS-R, visual memory	72	Significantly below average
WMS-R, long-term memory	56	Massively below average
VLMT	46 Learning, 6 interference, 11 and 6 in trials 6 + 7, and 42/7 in recognition	Below average
Doors test	A = 8 correct; B = 2 correct	Simple recognition (A) slightly below average, complex (B) severely below average
Emotional pictures test (recognition)	4 Errors	Normal
**RETROGRADE MEMORY**		
Famous faces test (38 pictures)	30 Directly recognized, 2 with cues	Above average
Autobiographical memory interview	Concrete remembrances reported, but few spontaneously	Subnormal
**PROCEDURAL MEMORY**		
Mirror reading task (two rounds)		Normal
**LANGUAGE**		
Boston naming test	All but 1 word identified	Above average
**EMOTIONS RECOGNITION AND EMOTION INTERPRETATION**		
Reading the mind in the eyes test	11/24 Correct	Below average
Multiple choice theory of mind test	8/16	Below average
**FLORIDA AFFECT BATTERY**		
Subtest 1 facial identity discrimination	11/20 Correct	Below average
Subtest 2 facial affect discrimination	14/20 Correct	Below average
Subtest 3 facial affect naming	13/20 Correct	Below average
Subtest 4 select facial affect	14/20 Correct	Below average
Subtest 5 facial affect matching	11/20 Correct	Below average
**PROBLEM SOLVING, COGNITIVE FLEXIBILITY, EXECUTIVE FUNCTIONS, RISK TAKING**		
Cronin-Golomb concept formation	16/17	Good average
Category test of Delis et al. (1992)	5, 4, and 3 categories	Below average
Tower of Hanoi (4 disks)	48 moves, 3 min, 1 s	Below average
Word fluency (controlled oral word test)	17 + 7 + 9	Below average (given his educational background)
Wisconsin card sorting test	27 Correct, 13 errors, 8 perseverative errors	Far below average
Game of dice task	With 18 moves −4400€ at end	No thoughtful strategy during first half of moves
**TENDENCY TO MALINGER**		
Rey 15-item test	All correct	No tendency of malingering
TOMM	First trial: 50/50	No tendency of malingering

### Laterality

SP was clearly lateralized to the right.

### Language and word knowledge

SP’s knowledge of terms and words, as assessed with the *Boston naming test*, was evidently above average (cf. Table 1). Given that a version of the *Boston naming test* had been administered several months prior to the assessment in our department, one might be inclined to attribute the results to a practicing effect. It is however noteworthy mentioning that the previously used variant only included 15 items. Furthermore, SP’s performance at that time had been deemed within normal limits.

### Attention, concentration, and processing speed

SP’s performance on tests of attention and concentration was non-uniform (cf. Table 1). In the Attention Index of the WMS-R he only gained 71 points. Similarly, in the *d2-test* his performance placed him in the lowest percentile. On the other hand, he achieved average scores in the *Trail Making Test A* and *B*. The results on *Trail Making Test A* and *B* were unexpected, given that a number of studies identified pronounced impairments in performance on these tests in patients with CADASIL without dementia and even without a history of stroke (Peters et al., 2005; Dichgans et al., 2008). Several factors might account for SP’s execution of the last two tests. Possibly, the task required here was more similar to what SP did in his professional life compared to the tasks in the other two tests; therefore he had probably more routine and subsequently made less errors. Another clarification might be the practicing effect (Bartels et al., 2010). Although SP did not recall or communicate that he had these tests before, reviewing the copies of reports revealed that he had been tested with the *Trail Making Test A* and *B* several months prior and had at that time achieved below average scores. Furthermore, there are indications that SP engaged in self-directed training of cognitive abilities at home (Smith et al., 2007).

### Intelligence and global cognitive performance

Several evaluation procedures were used for assessing SP’s intellectual abilities. The tests applied revealed that SP had a quite high verbal intelligence; however, his performance on both non-verbal parts of IQ testing and in tests that are used for preliminary investigation of the suspicion of dementia (*DemTect, MMSE*) was situated at low or borderline levels. As SP’s academic formation and profession required verbal skills and a broad vocabulary to a considerable extent, the high scores on verbal intelligence tests are not surprising (Denzler et al., 1986; Christensen et al., 1997; Parkin and Java, 1999). They point to a stability of measures of crystallized intelligence (Christensen et al., 1997) on a background of high educational achievement in this patient, in comparison to measures of fluid intelligence (Christensen et al., 1997; Manard et al., 2014). The scores obtained with instruments for short assessment of suspicion of dementia were however surprisingly low, given SP’s educational and occupational attainments. SP scored 27 on the *MMSE*. His performance on the *DemTect* [a global cognitive assessment instrument that includes measures for mental flexibility and verbal (category) fluency] was suggestive of “mild cognitive impairment” (cf. Table 1) (Peters et al., 2005). The discrepancy between the results on the two tests could be attributed to a larger degree of underestimation of executive dysfunction in *MMSE* (Benisty et al., 2008).

### Visual-constructive skills

Performance in copying the Rey–Osterrieth Figure was within normal limits (cf. Table 1). We did not find out evidence that he had taken part in this test before.

### Short-term memory and working memory

In the Wechsler Memory Scale subtests for short-term and working memory he was somewhat impaired. In digit span forward he was normal, being able to repeat five digits. In all other measures (digit span backward, block span forward, and backward) he only achieved four digits.

### Long-term memory

A large number of tests were applied to assess SP’s long-term memory abilities. They can be grouped into tests of anterograde, retrograde, episodic-autobiographical, semantic, procedural, and priming memory (cf. Markowitsch and Staniloiu, 2012, and Tulving, 2005).

#### Anterograde memory

**Figure 2:**
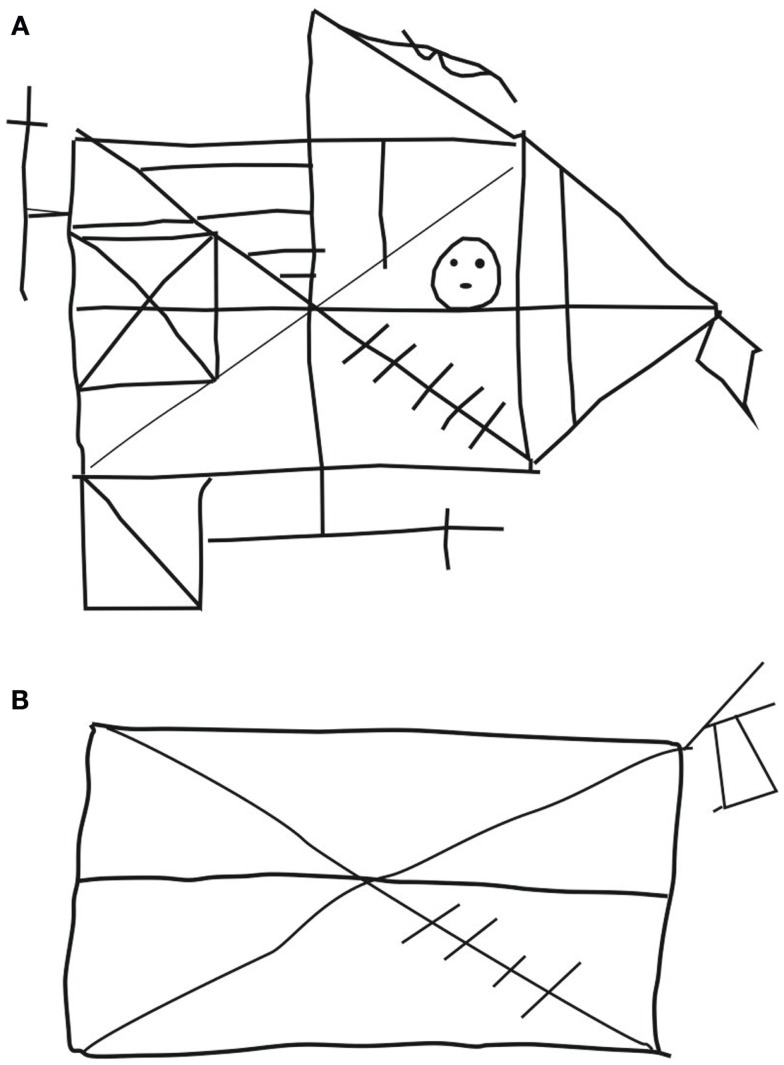
**Copying (A) and reproduction by heart after half an hour (B) of the Rey–Osterrieth figure**.

On all tests of anterograde memory – with the exception of two simple recognition tasks (*the less demanding section of the Doors test, Emotional Pictures Test*) – SP performed below the level of normal individuals. The finding of relatively preserved recognition in comparison to recall is congruent with other reports (Peters et al., 2005) (Figure 2). Verbal material was best retrieved (*VLMT, verbal memory index of the WMS-R*), while retrieval of visual material was the most compromised (*ROF, visual memory index of the WMS-R, complex part of the doors test*). Incidentally, these results are also consistent with other reports in the CADASIL literature (Amberla et al., 2004; Buffon et al., 2006). Interestingly, difficulties with retrieving visual material have also been reported in other conditions with lesions of fronto-striatal connections, such as Parkinson’s disease (Smith et al., 2010; Souchay et al., 2013). Savage et al. (1999) proposed that fronto-striatal dysfunctions might lead to impairments in retrieving non-verbal material (Rey–Osterrieth Complex Figure) due to an organizational deficit in the copy condition, related to problems with shifting mental or spatial sets. One might argue that a more favorable performance on verbal memory tasks might reflect SP’s background. Alternatively, it may bear connections with sub-clinical visual system impairments and partly with color perception abnormalities (Glen et al., 2012). SP’s performance was particularly poor when a delay of half an hour had to be bridged between learning and free recall (*ROF, long-term memory index of the WMS-R*) (cf. Table 1).

#### Retrograde memory

##### Retrograde semantic memory recognition

SP recognized famous persons very well (*famous faces test*) (cf. Table 1).

##### Retrograde autobiographical memory recall

SP spontaneously generated an impoverished account of specific details of autobiographical episodes from this past (*autobiographical memory interview*), resembling in this respect patients with a prodrome of Alzheimer’s dementia (Seidl et al., 2006). This finding might have been compounded in his case by the severe executive functions deficit (Brand and Markowitsch, 2008). Additionally, SP showed impaired retrieval of visual material. One might therefore argue that a compromised capacity to retrieve visual material might have also contributed to the production of schematic, overgeneralized autobiographical memories, with meager detail specificity (Bauer, 1982; Ogden, 1993; Greenberg et al., 2005; Smith et al., 2010; Souchay and Smith, 2013).

#### Procedural memory

SP’s procedural skills, as assessed by his capacity to carry out *mirror image reading* appeared to be intact (cf. Table 1), despite presence of damage in the basal ganglia. Intact performance on the mirror image reading might reflect not only the preserved ability to learn and store new procedural skills, but may be supported by various cognitive components, including high levels of verbal intelligence (Markowitsch and Härting, 1996). Incidentally, other authors found uncompromised performance on mirror reading in patients with various fronto-striatal lesions (Schmidtke et al., 2002).

#### Malingering

In the two instruments applied to investigate tendencies for malingering (Rey 15-Item-Test; TOMM), SP performed without any error and subsequently showed no tendency to malinger (cf. Table 1). We did not expect SP to feign impairments, but administered these tests to be on the safe side with respect to interpreting his performance in tests of memory.

#### Executive functions, cognitive flexibility, risk taking, and problem solving abilities

SP’s problem solving and executive abilities were largely impaired, with the only exceptions being the *TMT-B* and a test of *concept formation* (Cronin-Golomb et al., 1987a,b). We already commented above about the possible practicing effects on TMT-B test results, which may have led to improvements in performance over time. In the problem solving test of Cronin-Golomb et al. (1987a,b), SP was shown 17 sheets of paper; each leaf presented a drawing on the left side of the paper and three drawings on the right side (e.g., a crescent moon on the left side and a penguin, a woodpecker, and an owl on the right side). He had to tell which of the three drawings on the right side matches best the drawing on the left side. In this test, the correct answer can be given by simply excluding the more unlikely answers. In this way, the test resembles the more simple long-term memory tests, in which only recognition is required (as opposed to free recall).

In tests, where SP had to actively generate material or ideas – such as in the *California card sorting test* (Delis et al., 1992), the *tower of Hanoi*, the *word fluency* [*COW*], the *supermarket task*, and the *Wisconsin card sorting test* – his performance was impaired. In the *game of dice task*, SP behaved in strong contrast with his scholar background in business and economics. He showed no evidence of a sound or reasonable strategy about how to gain money in the fictitious game and consequently behaved randomly with respect to his choices. The findings of impairments in executive functions (planning, problem solving, set-shifting, cognitive flexibility, categorization, error detection, and monitoring) are in agreement with reports in the literature (Peters et al., 2005; Buffon et al., 2006). Contrary to other descriptions, we did not find evidence of relative preservation of performance on letter fluency tasks in comparison to category fluency tasks (Amberla et al., 2004; Dichgans, 2009).

#### Tests for social information processing (perception and interpretation of emotional states, theory of mind)

SP was impaired (partly even severely impaired) in tests of affect discrimination, naming, selection, and matching as well as facial identity discrimination [Florida (Tübingen) *Affect Battery* (*Subtests 1-*)]. *Tests for social cognition* (“Augen-ToM-Test” or *Reading the Mind in the Eyes Test* or RMET, *multiple choice theory of mind test or MCTT*) (cf. Table 1) yielded indices of below average performance as well. Both on Florida (Tübingen) Affect Battery subtests and “Augen-ToM-Test” SP encountered significant problems with the emotion of fear. Impairments in recognizing facial emotions (especially fear) have recently been reported in a group of patients with CADASIL by Valenti et al. (2013). In contrast to our patient, who showed no indication of depressive symptomatology, a substantial number of patients in the study of Valenti et al. (2013) endorsed depressive symptoms.

#### Tests for mood, personality, and psychopathological and psychological load screening

The screening instrument *Beck depression inventory* did not yield scores suggestive of an affective (depressive) disorder. The *symptom check list (SCL-90-R)* analysis did not generate abnormal scores. In the FPI-R, SP chose descriptors’ values suggesting reduced life satisfaction, self-focused attitude, self control, very little aggressiveness, and moderately reduced concern about health. He perceived himself as being mildly introverted and emotionally stable. He experienced himself as being highly passive and with decreased motivation for achievement. The latter finding is of interest, given a high percentage of patients with CADASIL experiencing apathy (Chabriat et al., 2009). We however did not identify any conspicuous evidence of apathetic behavior. On the openness subscale of the *FPI-R*, SP scored within normal limits. In the *self-evaluation questionnaire*, SP only gave partial responses with respect to changes since his CADASIL diagnosis. He indicated that he was very afraid of having to take a premature, forced retirement, and of losing his partner. He reported increased appetite and relinquishing some of his previous hobbies.

**Figure 3:**
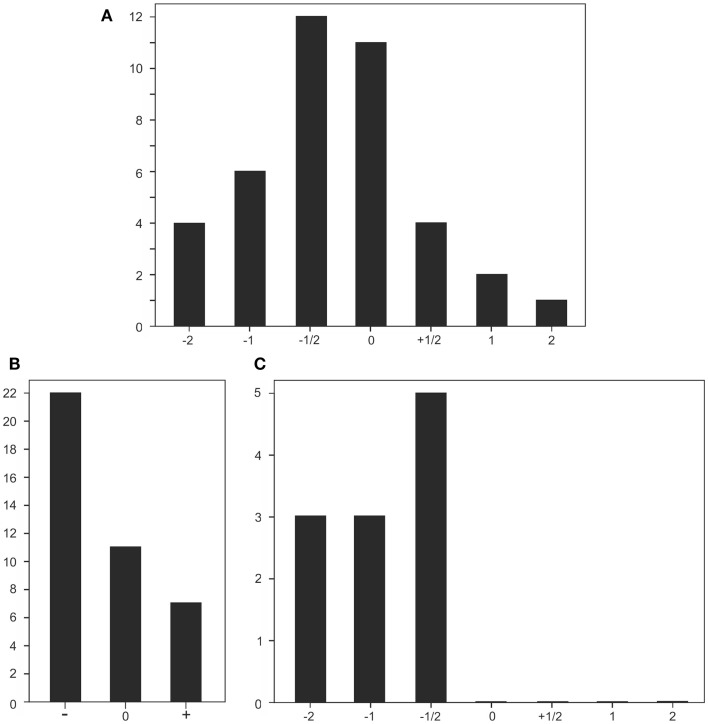
**Graphical illustrations of test results**. **(A)** Performance distribution in all 40 tests applied; scores roughly refer to standard deviations: 1/2 = less than a standard deviation below (−) or above (+) 0; ±1 or ±2 refer to about 1 or 2 standard deviations below or above baseline. **(B)** Performance distribution of tests with an inferior to normal (−), a normal (0), and a superior to normal performance (+). **(C)** Distribution of performance in tests measuring anterograde memory.

Figure 3 gives an overview of his test results, demonstrating that performance on a large array of the applied tests was on the deficient side. This is particularly evident in the illustration showing the distribution of inferior, normal, and superior test performance (Figure 3B), and especially for test results on anterograde memory, where his performance for all tests was situated on the inferior level (Figure 3C).

## Discussion

While the diagnosis of CADASIL is established (Hervé and Chabriat, 2010; Russell, 2010; Assareh et al., 2011; Yamamoto et al., 2011; Federico et al., 2012), case descriptions with a detailed neuropsychological examination identifying areas of deficiency and preservation can enrich the understanding of this condition and provide novel insights and ideas for larger scale research.

SP’s case features a constellation of unique characteristics in our opinion. The clinical manifestations have included less common symptoms and findings, such as seizures, hypertension, diplopia, sub-clinical involvement of optic nerve as well as of the nerve VIII (André, 2010; Yamamoto et al., 2011). The misleading clinical presentation, pattern of laboratory results, and the seemingly negative family history of CADASIL are also atypical traits, which have posed a substantial challenge to securing a definitive diagnosis. The pattern of laboratory results pointed to features of dysregulated immunity. These features may have been underpinned by a history of infection with *B. burgdorferi* (Zeman et al., 1993). Alternatively, they may reflect an unusual form of CADASIL, with inflammatory-like processes (Dichgans et al., 1999; Bentley et al., 2011; Collongues et al., 2012). SP’s distinctiveness might also pertain to his intellectual background and his profession. Another peculiar aspect is the progression of his cognitive functioning, encompassing marked deteriorations in a large number of cognitive domains being documented shortly after the diagnosis of CADASIL, limited insight into cognitive problems and reasonably maintained ability to put forth a façade of preserved intellectual functioning. SP did not endorse symptoms of depression and did not meet criteria for other psychiatric disorders. The bulk of neuropsychological examination results stayed in strong contradiction with his own appraisals of his cognitive difficulties. Furthermore, SP’s structural imaging indicated substantial brain damage, involving subcortical structures, white matter as well as cortical areas.

Interestingly, SP’s “crystallized” (verbally based) intelligence was still outstanding in one test and above average in another. This finding is in opposition to the results of Matsuda and Saito (1998) who in elderly patients with mild Alzheimer’s dementia found deficits to be stronger in crystallized intelligence in comparison to age-matched healthy controls, while “fluid” intelligence measures did not deviate significantly from those of healthy participants. However, SP showed severely compromised performance on a number of intellectual measurements. In this way he resembled the three patients studied by Dominguez-Sanchez et al. (2011), who all lost a significant intelligence quotient and general intellectual capacity during the progression of CADASIL (7 years follow-up).

In the memory domain, SP showed a number of impairments. His subaverage performance in short-term memory and working memory tasks likely bears relations to changes in parietal and prefrontal areas (Markowitsch et al., 1999) and in frontal–subcortical circuits (Amberla et al., 2004). Massive impairments were noted on tests of anterograde (conscious) long-term memory. Similarly to his crystallized intelligence, his old knowledge was largely maintained. SP was still very able to recognize and name portraits of prominent persons (*famous faces test*). However, when he had to actively generate episodes of his own life, SP provided an impoverished, rudimentary, semanticized account.

SP’s deficits with consciously acquiring mnemonic information for long-term storage could have stemmed from his damage to temporal as well as parietal areas and fiber connections (Markowitsch and Staniloiu, 2012; Staniloiu and Markowitsch, 2012). There are numerous meticulous documentations of anterograde memory impairments after bilateral damage of medio-temporal regions. Furthermore, studies show that hippocampal damage might be a route of impairment in anterograde conscious memory in CADASIL (as well as in patients with seizures) (Jokeit et al., 1997; O’Sullivan et al., 2009; Markowitsch and Staniloiu, 2012). Damage to the thalamus and diencephalic tracts may constitute another source of anterograde memory impairments in patients with CADASIL (Markowitsch, 1991; O’Sullivan et al., 2004; Chabriat et al., 2009; Markowitsch and Staniloiu, 2012). Interestingly, evidence of microbleeds in the thalamus was reported in the case of SP. Additionally, damage within prefrontal regions may also partly contribute to impairments in the acquisition of semantic and episodic mnemonic information for long-term storage (Rieger and Markowitsch, 1996; Markowitsch and Staniloiu, 2012).

As SP’s ability for long-term concentration was impaired as well (particularly evident from the multiple omissions in the *d2-Test*), this might have added to both his anterograde and retrograde memory impairments. The attentional deficits may have stemmed from damage in parietal and frontal areas and their connections, and possibly from diencephalic and brainstem lesions (Mesulam, 1990). Parietal regions have been implicated not only in attentional processes, but also in short-term memory, episodic memory, self referential processing, and time perception (Babinsky et al., 1996; Beblo et al., 2001; Cabeza et al., 2011). They are opined to be engaged in modulation of top-down (frontal) and bottom-up (medial temporal lobe areas) episodic memory-related processes (Beblo et al., 2001; Cabeza et al., 2011; Souchay et al., 2013). The impairments in retrograde episodic-autobiographical memory observed in SP might deliver support for the idea that intact right fronto-temporal connections are important for “ecphorizing” old, emotionally tagged events (Fink et al., 1996; Kroll et al., 1997). As mentioned previously, SP had lacunar defects in the right temporal lobe and adjacent to the right amygdala as well as gliotic changes affecting both anterior temporal lobes.

Consistent with established descriptions in the literature, SP showed a pronounced executive dysfunction syndrome. Planning functions and the ability to overview necessary steps in solving more complex tasks were particularly deficient. This cannot only be inferred from SP’s severe impairments in the *Wisconsin card sorting test* and *California card sorting test*, but also from the way he performed in the *d2-Test* where he should have crossed out all letters “d” which were accompanied by two small lines above or below them. He had a very high number of omissions, being apparently simply focused to reach the end of each of the 14 long letter lines – an aim usually impossible to accomplish.

A striking finding was the large extent of deficits in face-emotion processing tests and theory of mind tasks. In the Florida Affect Battery (Tübingen Affect Battery), SP’s performance was compromised on both simple and complex tasks. These results might reflect damage of cortical and subcortical areas (and their fiber connections), which are recruited in face and emotion processing (Herholz et al., 2001; Vuilleumier et al., 2004; Markowitsch and Staniloiu, 2011). As mentioned above, in the case of SP we visualized lacunar defects in the right temporal lobe and in regions neighboring the right amygdala. Amygdalar dysfunction and/or damage of anterior hippocampus could lead to impairments of fear processing (Fanselow and Dong, 2010; Feinstein et al., 2011). The mentalizing deficits of SP might have originated from disruptions of temporal, parietal, and frontal regions and their connections (Abu-Akel, 2003) as well as damage of basal ganglia (Bodden et al., 2010; Kemp et al., 2013).

SP’s apparently reduced insight and awareness into his cognitive deficits (especially in the memory domain) could have both biological and psychological underpinnings (Naylor and Clare, 2008; André, 2010). From a neuropsychological point of view, the diminished insight may mirror a collapsing executive system, faulty self knowledge updating processes, and impaired self awareness (Naylor and Clare, 2008; Klein and Gangi, 2010). With respect to neural correlates, these insight deficits may be underlain by the observed changes in fronto-parietal regions (Wagner et al., 2005; Stone et al., 2006; Metzinger, 2008; Nyberg et al., 2010; Souchay et al., 2013). From a psychological perspective, the deficient insight may reflect a desire to mask or deny his difficulties, in order to sustain a positive sense of identity (Naylor and Clare, 2008).

## Conclusion

The present case report highlights a number of new as well as more established facets of CADASIL.

The most striking and novel aspect of our case is the extent of impairments in the episodic-autobiographical domain and social information processing (face-emotion processing, social cognition). These changes had not been documented to these degrees in patients with CADASIL. Whether these identified impairments are related to the patient’s specific phenotype (e.g., history of seizures, markers of dysregulated immunity) or to an ascertainment bias (e.g., a paucity of studies investigating these cognitive functions) will need to be clarified by larger scale research. The systematic investigation of social information processing in CADASIL patients is important as deficits in this domain might constitute hidden and often unrecognized and neglected sources of occupational and interpersonal difficulties. Although the informant history did not provide a clear corroboration of SP’s deficits in social information processing, it is possible that at least some of his occupational and interpersonal difficulties might have been underpinned by his problems with social information processing.

Our case furthermore underlines the large phenotypic variability of CADASIL, which may be shaped by gene-environment interplays (Yamamoto et al., 2011). Although rarely reported, presentations with markers of dysregulated immunity might occur, as our case points to. The dysregulated immunity might reflect the presence of a concurrent infectious or immune condition, or a gene-environmental interplay (Bentley et al., 2011). These aspects have possible implications for the differential diagnosis and for the exploration of immune-modulating treatment approaches.

Finally, our case description suggests that the cognitive decline caused by CADASIL may follow a differentiated trajectory in patients with high intellectual and educational attainment in comparison to those with lower levels of educational and occupational achievement. In this respect CADASIL may have a similar impact on cognition as AD – though usually starting much earlier in life. For AD it has been repeatedly found that individuals starting from a high intellectual background will deteriorate faster and more profoundly than those starting from a moderate intellectual background (Scarmeas et al., 2006; Wilson et al., 2010). A high degree of cognitive reserve in patients with CADASIL might initially act as a buffer against the consequences of brain changes, braking the cognitive decline. When a critical burden of brain lesions is reached, this adaptation mechanism might break down, unraveling the consequences of brain damage and apparently getting translated into a more rapid cognitive decline in these patients in comparison to patients with a lower cognitive reserve (Perneczky et al., 2009). Though no firm conclusions can be drawn from studying a single case, our case report joins proposals for extending and making use of the concept of cognitive reserve for the understanding of vascular cognitive deficits (Stern, 2002; Zieren et al., 2013).

## Conflict of Interest Statement

The authors declare that the research was conducted in the absence of any commercial or financial relationships that could be construed as a potential conflict of interest.
